# Targeted Therapy of FLT3 in Treatment of AML—Current Status and Future Directions

**DOI:** 10.3390/jcm3041466

**Published:** 2014-12-15

**Authors:** Caroline Benedicte Nitter Engen, Line Wergeland, Jørn Skavland, Bjørn Tore Gjertsen

**Affiliations:** 1 Center for Cancer Biomarkers CCBIO, Department of Clinical Science, University of Bergen, Bergen N-5020, Norway; E-Mails: caroline.engen@k2.uib.no (C.B.N.E.); line@wergeland.jacobsen.net (L.W.); jorn.skavland@k2.uib.no (J.S.); 2 Department of Internal Medicine, Hematology Section, Haukeland University Hospital, Bergen N-5021, Norway

**Keywords:** acute myeloid leukemia, FLT3, tyrosine kinase inhibitors, clinical trials

## Abstract

Internal tandem duplications (ITDs) of the gene encoding the Fms-Like Tyrosine kinase-3 (FLT3) receptor are present in approximately 25% of patients with acute myeloid leukemia (AML). The mutation is associated with poor prognosis, and the aberrant protein product has been hypothesized as an attractive therapeutic target. Various tyrosine kinase inhibitors (TKIs) have been developed targeting FLT3, but in spite of initial optimism the first generation TKIs tested in clinical studies generally induce only partial and transient hematological responses. The limited treatment efficacy generally observed may be explained by numerous factors; extensively pretreated and high risk cohorts, suboptimal pharmacodynamic and pharmacokinetic properties of the compounds, acquired TKI resistance, or the possible fact that inhibition of mutated FLT3 alone is not sufficient to avoid disease progression. The second-generation agent quizartinb is showing promising outcomes and seems better tolerated and with less toxic effects than traditional chemotherapeutic agents. Therefore, new generations of TKIs might be feasible for use in combination therapy or in a salvage setting in selected patients. Here, we sum up experiences so far, and we discuss the future outlook of targeting dysregulated FLT3 signaling in the treatment of AML.

## 1. Introduction

### 1.1. Acute Myeloid Leukemia

Acute myeloid leukemia (AML) is the most frequent acute leukemia in adults [1,2]. It is a heterogeneous clonal disorder of the myeloid precursor cells [3], and although patients today are treated with similar nonspecific treatment regimens that have remained more or less unchanged for decades [4,5,6], it has for an equally long period been recognized that there is considerable genetic, biological, and clinical heterogeneity in the patient group [7,8]. This variation is clearly reflected in the diverging relapse rate and overall survival in response to standard of care, ranging from 10%–70%, dependent on both patient and disease related factors [9,10]. Current risk stratification at the time of diagnosis usually include age, performance status, white blood cell count, determining if the disease is *de novo*, secondary or therapy related, cytogenetics, and mutation analysis [11]. Several cytogenetic [12,13], molecular genetic (e.g., Fms-Like Tyrosine kinase-3 (FLT3), nucleophosmin 1 (NPM1), CCAAT enhancer-binding protein-α (CEBPA)) [14], and epigenetic changes [15], as well as aberrantly expressed RNA, and microRNA [16] have been identified as prognostic markers for disease outcome, and as shown in other hematological malignancies it is thought that some of these changes represent feasible therapy targets. The challenge we are facing today is to translate this knowledge into tailored treatment for AML, identifying and directing the treatment towards cancer-specific pathways aiming for improved patient outcome.

### 1.2. Mutations and Signaling Pathways in AML

Normal hematopoiesis is controlled by the microenvironment and external signaling molecules, transmitting signals through intracellular signal transduction pathways via cell surface receptors. These intracellular pathways form a highly complex network of signaling cascades, including receptors, kinases, phosphatases and transcription factors that cross-talk extensively on multiple levels. Changes in this network by cytogenetic abnormalities, mutations or epigenetic alterations may lead to non-functional or hyper-activated pathways, that in turn can lead to anti-apoptosis and increased proliferation of the cells [17,18].

The group of genes most frequently mutated in AML is signaling genes, including genes coding for receptor tyrosine kinases such as FLT3 and KIT, Serine-Threonine kinases, KRAS/NRAS and protein tyrosine phosphatases [19]. Aberrant regulation of intracellular signaling pathways accordingly appears to be an important leukemia promoting mechanism, and like inhibition of Bcr-Abl revolutionized patient outcome in chronic myeloid leukemia [20], targeting signaling onco-proteins seems like a feasible strategy in AML [21]. 

The one most frequent mutated gene in AML, with mutations detected in up to 35% of the patients, is the Fms-Like Tyrosine kinase-3 (FLT3) gene on chromosome 13q12. Two major classes of FLT3 mutations have been identified: length mutations, predominantly internal tandem duplications (ITD) in the juxtamembrane domain of FLT3, first described by Nakao *et al*. in 1996 [22], and tyrosine kinase domain (TKD) point mutations [23,24]. ITDs are detected in 20%–25% of AML patients, while about 5%–10% of patients have point mutations within the TKD, with a mutation at codon 835 being the most frequent one [22,23,24].

### 1.3. Aberrant FLT3 Activation in AML

FLT3 is a member of the tyrosine kinase III family and functions as a membrane bound growth factor receptor, usually expressed by human hematopoietic progenitor cells [25]. Binding of its ligand, FLT3-ligand (FL) induces a conformational change in the protein that causes activation of the intrinsic tyrosine kinase domain. The enzyme phosphorylates intracellular molecules and consequently activates multiple downstream signaling pathways involved in cellular survival, proliferation and differentiation [26]. The expression of FLT3 is normally lost upon differentiation [27,28], but as AML is caused by a block in differentiation and uncontrolled proliferation of the myeloid progenitor cells the expression is frequently “captured” in many AML blasts, and remains highly expressed in most AML cases [29,30]. While overexpression of the receptor has been associated with poor prognosis [31], the presence of FLT3-ITD mutations confers even stronger independent prognostic information as it significantly correlates with an increased risk of relapse and dismal overall survival, in comparison to the TKD mutation where such an associations is absent [32,33,34]. Although FLT3-ITD positive AML is not considered a distinct entity of AML, FLT3-ITD status has been included in the WHO 2008 guidelines and the European LeukemiaNet recommendations for classification of AML, providing important prognostic information [11,35]. The survival advantage of leukemic blasts driven by mutant FLT3 is to a large extent thought to be explained by a constitutive activation of the receptor causing FL independent autophosphorylation [36], and initiation of two major intracellular pathways essential for growth, survival and proliferation; PI3K/AKT/mTOR and RAS/RAF/MEK/ERK [37]. The signal transducers and activators of transcription (STATs) are usually not regulated via RTKs, but for mutations like FLT3-ITD a constitutive phosphorylation and transcriptional activation of STAT5 also occur [38,39]. 

With aberrant signaling appearing as a key component in FLT3-ITD mutated AML the constitutive active surface protein stands out as an attractive target for small molecule receptor inhibitor-based therapy [40,41]. Over the 15 years since the discovery of the mutated receptor and its clinical significance, more than a dozen different tyrosine kinase inhibitors (TKI) have been developed and tested preclinically, and many have shown to selectively induce cell death in FLT3 mutated AML blasts by suppressing FLT3 autophosphorylation and downstream signaling pathways [40,41,42]. Several of the agents, including the first generation agents lestaurtinib, linifanib, midostaurin, semaxanib, sorafenib, sunitinib, and tandutinib as well as the second generation agent quizartinib, have reached clinical trials where their safety, tolerability, and efficiency have been assessed. In the following section we will discuss and compare the most relevant FLT3 TKIs in clinical trials. Both trials where the TKIs are used as monotherapy and trials where conventional treatment is combined with a TKI will be assessed and summarized, with focus on antileukemic efficacy and side effects (Table 1).

**Table 1:** Overview of evaluated clinical trials.

Agent	Study Phase	Patient Population	*n*	Median/Mean Age (years)	FLT3-ITD	FLT3-Point-Mutation Only	Treatment	Dose	Ref.
Lestaurtinib—CEP-701	Phase 1/2	AML, refractory/relapsed	17	61 (18–71)	94.1% (*n* = 16)	5.9% (*n* = 1)	Monotherapy	40 mg–80 mg × 2	[43]
	Phase 2	AML, untreated	29	73 (67–82)	6.9% (*n* = 2)	10.3% (*n* = 3)	Monotherapy	60 mg–80 mg × 2	[44]
	Phase 2 (Randomized)	AML, first relapse	224	56.5 (20–81)	92% (*n* = 206)	7.6% (*n* = 17)	+ Mitoxantrone, Etopside & Cytarabine	80 mg × 2,	[45]
Linifanib—ABT-869	Phase 1	AML, refractory/relapsed	47	56.3 (23–81)	12.8% (*n* = 6)	10.6% (*n* = 5)	Monotherapy/+ Cytarabine	5–25 mg	[46]
Midostaurin—PKC412	Phase 2	AML, refractory/relapsed, High risk MDS	20	62 (29–78)	90% (*n* = 18)	10% (*n* = 2)	Monotherapy	75 mg × 3	[47]
	Phase 2B	AML, refractory/relapsed, High risk MDS	95	64% ≥ 65 years	27.4% (*n* = 26)	9.5% (*n* = 9)	Monotherapy	50 mg–100 mg × 2	[48]
	Phase IB	AML, untreated	69	48.5	17.4% (*n* = 12)	8.7% (*n* = 6)	+ Daunorubicin & Cytarabine	50 mg–100 mg × 2	[49]
Semaxanib—SU5416	Phase 2	AML, refractory or advanced, High risk MDS	33	64 (23–76)	4.5% (*n* = 1/22)	NA	Monotherapy	145 mg/m^2^, twice weekly	[50]
	Phase 2	AML advanced, c-kit pos.	43	65 (27–79)	20% (*n* = 7/35)	NA	Monotherapy	145 mg/m^2^, twice weekly	[51]
	Phase 2	AML refractory, High risk MDS	55	64–66 (22–80)	NA	NA	Monotherapy	145 mg/m^2^, twice weekly	[52]
Sorafanib—BAY 43-9006	Phase 1	AML, refractory/relapsed	16	61.5 (48–81)	43.8% (*n* = 7)	12.5% (*n* = 2)	Monotherapy	200 mg–600 mg × 2	[53]
	Phase 1	AML refractory/relapsed, High risk MDS	42	71.3	33% (*n* = 9/27)	NA	Monotherapy	100 mg–400 mg × 2	[54]
	Phase 2 (Randomized)	AML, >60 years	197	68 (61–80)	14%	NA	+ Cytarabin and Daunorubicun	400 mg × 2	[55]
	Phase 1	Acute leukemia, refractory/relapsed	12	9.5 (6–17)	41.7% (*n* = 5)	NA	+ Clofarabine & Cytarabine	150 mg/m^2^/200 mg/m^2^ × 2	[56]
	Phase 1/2	AML, refractory/relapsed	43	64 (24–87)	93% (*n* = 40)	NA	+ 5-Azacytidine	400 mg × 2	[57]
Sunitinib—SU11248	Phase 1	AML	29	67 (19–82)	10.3% (*n* = 3)	6.9% (*n* = 2)	Monotherapy	50 mg–350 mg as a single dose	[58]
	Phase 1	AML, refractory	15	72 (54–80)	14.3% (*n* = 2/14)	14.3% (*n* = 2/14)	Monotherapy	50 mg–75 mg	[59]
Tandutinib—MLN-518	Phase 1	AML, High-risk MDS	40	70.5 (22–90)	20% (*n* = 8)	2.5% (*n* = 1)	Monotherapy	50 mg–700 mg × 2	[60]
Quizartinib—AC220	Phase 1	AML	76	60 (23–83)	27% (*n* = 18/65)	NA	Monotherapy	12–450 mg × 1	[61]
	Phase 2	AML, refractory/relapse	76	53 (19–77)	100% (*n* = 76)	NA	Monotherapy	30–60 mg	[62]
	Phase 2	AML, refractory/relapse, unfit	270	60.4 (19–85)	70.7% (*n* = 191)	NA	Monotherapy	90–135 mg	[63,64]
	Phase 1	AML, untreated >60 years old	55	69 (62–87)	7.3% (*n* = 4)	NA	+ Cytarabin, Daunorubicin & Etoposide	40–135 mg	[65]
	Phase 1	AML, MLL-rearranged ALL, >1 month, ≤21 years	22	NA	27.3% (*n* = 6)	NA	+ Cytarabin & Etoposide	25–60 mg/m^2^	[66]

## 2. Evaluation of Selected Small Molecule Inhibitors against FLT3 Used in Clinical Trials

### 2.1. First Generation TKIs

#### 2.1.1. Lestaurtinib (CEP-701)

Lestaurtinib is an orally bioavailable polyaromatic inolocarbazole alkoid compound that is synthetically derived from the bacterial fermentation product K-252a. It was originally identified as an inhibitor of the neurotropin receptor TrkA, and was initially studied in patients with solid tumors [42]. It has successively been found to be a potent FLT3 inhibitor, and has been investigated in AML patients [43,44,45]. In a phase 1/2 trial FLT3-mutated patients with advanced AML the drug was found to be generally well tolerated; with observed treatment related toxicities including mild nausea and emesis, and generalized weakness and fatigue. Clinical activity was observed in 29% of the patients during a limited time period, ranging from two weeks to three months. The drug significantly lowered peripheral blood blasts, and some patients had evidence of transient normal hematopoiesis [43]. In a phase 2 trial, lestaurtinib was administered in monotherapy as first-line treatment in 29 older AML patients not considered eligible for intensive chemotherapy. The drug was given for eight weeks, regardless of FLT3-mutation status. Observed toxicities included mild gastrointestinal side effects. No complete or partial remissions were seen, but transient reduction in bone marrow and peripheral-blood blasts was achieved in 60% (3/5) of the FLT3-mutated patients, compared to a 22.7% (5/22) response rate in the FLT3-wild-type group. The clinical response was however of short duration, with a median time to progression of 25 days [44]. In a bigger randomized phase 2 trial, 220 FLT3 mutated AML patients at first relapse received either chemotherapy alone or chemotherapy followed by lestaurtinib. There was no significant difference in the rate of adverse effects in the two groups, however, the seriousness of adverse effects was higher in the lestaurtinib-treated group. Of the patients receiving lestaurtinib 25.9% (29/112) patients achieved complete remission or complete remission with incomplete platelet recovery, compared to 20.5% (23/112) patients attaining equal treatment responses in the control group. There was however no significant difference in overall survival between the two groups, providing no clear benefit to adult AML patients with FLT3 mutations [45]. 

#### 2.1.2. Linifanib (ABT-869)

Linifanib is an orally available potent inhibitor of FLT3 and VEGFR. Preclinically it has shown antileukemic effects both as monotherapy and in combination with cytarabine in FLT3-mutated human AML xenograft models [67]. In a phase 1 dose-escalation study, relapsed or refractory AML patients were treated either with linifanib alone or linifanib in combination with intermediate-dose cytarabine. Generally linifanib was well tolerated, and the most common side effects related to the treatment were fatigue, gastrointestinal distress and infections. The primary objective in the study did not include efficacy, but antileukemic effects were observed both in patients with FLT3 mutated as well as FLT3 wild-type patients [46]. 

#### 2.1.3. Midostaurin (PKC412, *N*-Benzoylstaurosporin)

Midostaurin is a derivate of staurosporine, initially developed as a protein kinase C inhibitor, and extensively used as model agent for the study of apoptosis. It is a multi-targeting TKI, inhibiting tyrosine kinases such as c-Kit and PDGFR as well as FLT3 [68]. In a phase 2 trial FLT3 mutated patients with relapsed or refractory AML or high-risk MDS not considered candidates for chemotherapy, were treated with midostaurin in monotherapy. The drug was generally well tolerated with the most frequent treatment related adverse effect being nausea and vomiting. The drug showed some transient clinical activity, reducing the amount of peripheral blasts by 50% in 70% (14/20) of the patients, and reducing bone marrow blast counts by 50% in 30% (6/20) of the patients [47,69]. In a larger phase 2B trial 95 AML/high risk MDS patients were randomized to receive either 50 mg or 100 mg of oral midostaurin twice daily, independently of FLT3 mutation status. Midostaturin was generally well tolerated in both concentrations and there were no clear difference in results according to dose regime. Side effects included nausea and vomiting. In the 92 patients, treatment efficiency could be assessed the reduction in peripheral blood or bone marrow blasts by 50% or more was 71% in the FLT3-mutated group compared to 42% in the FLT3-wild-type group. The majority of patients with FLT3-mutations responded with a reduction in blast count. One partial response was seen in a FLT3-ITD positive patient at the 100 mg per day regime. Hematological improvement was seen in 46% of the patients with FLT3-ITD versus 35% of the FLT3-wild-type patients. All therapy naive FLT3-ITD patients had a clear reduction of peripheral blood and bone marrow blasts [48]. In a phase 1B study, 69 younger newly diagnosed AML patients were treated with midostaurin in addition to a standard of care regime consisting of daunorubicin and cytarabine. The treatment cycle run for 28 days with one group getting the inhibitor concomitant starting on day 1–7 and day 15–21 and a second group getting the inhibitor administered sequentially starting on day 8–21 with 14 treatment days per cycle. The first 29 patients received midostaurin 100 mg orally twice daily. This dosage regime was discontinued because of adverse effects and followed by 40 AML patients who received midostaurin 50 mg twice daily. The treatment at the 50 mg twice-daily regime was generally well tolerated. The complete remission rate of the patients in the FLT mutant group (*n* = 18) and wild-type group (*n* = 51) was 92% and 74%, respectively [49]. Initial results from a phase 1/2 study of midostaurin and 5-Azacytinine in combination in refractory or relapsed AML demonstrates that it is a feasible alternative with a complete remission rate of 25%, and additionally 20% of patients achieving complete remission with incomplete platelet recovery [70]. An additional phase 1 study of midostaurin, bortezomib and chemotherapy shows promising antileukemic activity in refractory/relapsed AML patients and further investigation is ongoing [71]. A larger placebo controlled phase 3 trial (ClinicalTrials.gov identifier: NCT00651261), comparing midostaurin in addition to standard induction therapy is currently in completion and may indicate the pathway forward for midostaurin in AML treatment.

#### 2.1.4. Semaxanib (SU5416)

Semaxanib is an indolinone derivate that inhibits VEGFR, c-Kit and FLT3. It produces a dose dependent inhibition of tumor progress in a diversity of xenograft models, comprising malignant melanoma, glioma, fibrosarcoma and carcinomas of the lung, breast, prostate, and the skin [72]. In a phase 2 study of 33 patients, either with refractory AML or advanced MDS, the effect of semaxanib in monotherapy was assessed. Semaxanib was infused intravenously twice weekly in a dose of 145 mg/m^2^. Objective responses were seen in 18.2% (4/22) of the AML patients; three patients attained a partial response while one patient achieved a hematologic improvement [50]. In a second phase 2 trial, c-Kit positive patients with advanced AML were treated with semaxanib in monotherapy. Observed toxicities included nausea, musculoskeletal pain, headache, insomnia, vomiting, vertigo, fatigue/malaise, abdominal pain, sweating, and arthralgia. Half of the patients included in the study experienced severe adverse effects, with pneumonia and sepsis being the most frequent. Of the 25 patients evaluable for clinical response, no remissions were observed among the FLT3-ITD positive patients (*n* = 7). One patient achieved a morphologic remission while 28% (7/25) patients experienced a transient partial response with at least 50% reduction in bone marrow and peripheral blood blasts. The mean response duration of all eight responding patients was 1.6 months until disease progression. Patients with AML blasts expressing high levels of VEGF mRNA had a significantly higher response rate compared to the rest of the patient group, indicating that the main antileukemic effect was mediated by semaxanib’s antiangiogenic properties rather than direct growth inhibition [51]. In a third phase 2 trial, 55 patients with refractory AML or advanced MDS were treated with semaxanib in monotherapy. Observed toxicities included headache, dyspnea, fatigue, thromboembolic events, bone pain and gastrointestinal events. Objective responses were obtained in 7.3% of the patients; three patients achieved partial responses and one patient experienced a hematologic improvement [52].

#### 2.1.5. Sorafenib (BAY 43-9006)

Sorafenib is an orally available bi-aryl urea that inhibits several kinases, including RAF-kinase, VEGFR-2, c-Kit, and FLT3. It is currently approved for the treatment of metastatic renal cancer and advanced hepatocellular carcinoma [73]. In a phase 1 trial, refractory or relapsed AML patients were treated with sorafenib 200 mg twice daily. A clinical response was seen in 56.3% (9/16) of the patients, including all of the FLT3-ITD positive patients (*n* = 6). Both circulating and bone marrow blasts were strongly reduced in patients with FLT3-ITD mutation, while there was no essential change in the patients without FLT3-ITD [53]. In a randomized phase 1 clinical and biologic study of sorafenib, 42 patients with either AML or MDS were randomized either to continuously administration of the drug, or intermittent. The drug was administered twice daily, and the dose increased during the trial to evaluate dose-limiting toxicity. Of the patients assessed 33% (9/27) were FLT3-ITD positive. Dose-limiting toxicity was prevalent at the 400 mg twice-daily regime. The most seen drug related side effects were of gastrointestinal character, including abdominal pain, nausea, and vomiting. Palmar-plantar dysesthesia among other toxic skin reactions were also seen. Three patients experienced arterial thrombosis; myocardial infarction, brain stem infarction and splenic infarcts. One complete remission, lasting 2.7 months, was observed in a FLT3-ITD positive patient. In 33.3% of the FLT3-ITD positive patients, an improvement in peripheral blood and bone marrow blast counts was observed [54]. Recently, the results from a randomized, placebo-controlled trial concluded that the combination of standard induction treatment with sorafenib as consolidating treatment was of no benefit for AML patients older than 60 years of age compared to standard induction therapy alone. On the contrary, this combination seemed to cause worse outcomes with more adverse effects. Event-free survival and overall survival was not significantly improved, and these results were consistent also in the FLT3-ITD positive subgroup of patients [55]. Initial results from a similar study however indicated that the addition of sorafenib to standard chemotherapy is associated with a high rate of complete remission and an acceptable toxicity profile in FLT3-mutated older AML patients [74]. In a phase 1 pharmacokinetic and pharmacodynamic trial sorafenib was studied in concurrence with the cytotoxic agents clofarabine and cytarabine in pediatric acute myeloid leukemia patients, who all had either relapsed or refractory disease. All patients experienced hand-foot skin reactions and/or rash, which was also the dose-limiting toxicities, with maximum tolerated dose determined to 150 mg/m^2^ twice daily. On day 8, sorafenib decreased blast percentages in 83.3% (10/12) of the patients. After combination chemotherapy three of five patients with FLT3-ITD mutations and three FLT3-wild-type patients achieved complete remissions. One additional FLT3-wild-type patient with AML attained a partial remission [56]. A retrospective assessment of FLT3-ITD positive pediatric patients suggested that post-transplant therapy with sorafenib might also improve outcome in patients that have been treated with hematopoietic stem cell transplantation [75]. In a phase 2 trial, 43 AML patients, mainly FLT3-ITD positive (93%), were treated with sorafenib in combination with 5-Azacytidine. Antileukemic efficacy was observed in 46% of the assessable patients, including a 27% complete remission rate [57]. 

#### 2.1.6. Sunitinib (SU11248) 

Sunitinib is an oral multi-targeting TKI, predominantly targeting PDGFR, VEGFR, c-Kit and FLT3. It has been used in treatment for multiple solid malignancies, and is approved for the treatment of metastatic renal cell carcinoma and gastrointestinal stromal tumors [76]. In a phase 1 clinical trial, 29 AML patients were treated with a single dose of sunitinib. The dose was escalated in 50 mg increments from 50 mg to a highest dose of 350 mg. Adverse effects occurred in 31% of patients reported at the 250–350 mg dose levels. The toxicities were mainly of mild gastrointestinal character, like diarrhea and nausea. Determination of clinical response was not a study target and was not assessed thoroughly. Peripheral blood blast counts were however analyzed at 24 and 48 hours after treatment and five patients exhibited a large decrease in blast count. Of these five patients, two had an FLT3-ITD mutation [58]. In a phase 1 study, 15 patients with refractory or resistant AML or patients not amenable for conventional therapy were treated with sunitinib in 4-week cycles at the starting dose of 50 mg, followed by 75 mg. In total, 33.3% of the patients were FLT3-mutated. Treatment related side effects were generally of mild character. At the 50 mg treatment regime three patients experienced grade 2 adverse effects. One patient experienced lower limbs edema and fatigue, a second patient experienced taste disturbances and dry skin, and a third patient experienced fatigue, nausea and vomiting, tenesmus, mouth ulcerations, gingivitis, circulation disorders, hematuria, proteinuria and increased creatinine. Both patients treated with 75 mg experienced dose limiting side effects. Forty percent of the patients experienced transitory morphologic or partial responses with reduction of the percentage of leukemic blasts in peripheral blood and bone marrow, including 100% of the patients within the FLT3-mutated group in comparison with 20% of the FLT3 wild-type patients [59].

#### 2.1.7. Tandutinib (MLN-518)

Tandutinib is a piperazinyl quiazoline type III TKI with very limited inhibition of kinases outside this receptor family of FLT3, PDGFR and KIT [77]. In a phase 1 study, 40 patients were given tandutinib orally in doses ranging from 50 mg to 700 mg twice daily. The patients had either AML, or high-risk MDS. The most frequent toxicities associated with tandutinib treatment were nausea and vomiting, less frequent diarrhea and peripheral and periorbital edema. Muscular weakness and fatigue were the dose limiting toxicities, and were observed at dose levels of 525 mg and 700 mg twice daily. One patient experienced hyperreflexia with clonus. Preclinical evaluation of tandutinib suggested that it might prolong the QT interval, and one patient had a 270 ms increase of QT_c_ on day 28. The QT interval however returned within normal range during continuous dosing. No complete or partial remissions were seen in this study. However, of the five patients with FLT3-ITD mutations that were assessed for treatment efficacy, antileukemic activity was shown in two patients. They both had a greater than 99% decrease in absolute peripheral blast count and a decrease in bone marrow blast percentage from 91% to 62% and 80% to 15% over the first 28 days of treatment. Within two months however they both experienced disease progression. Four patients without FLT3-ITD mutation sustained steady peripheral blood counts and bone marrow blast counts in periods ranging from 154–190 days [60].

### 2.2. Second Generation TKIs

#### Quizartinib (AC220)

Quizartinib is unique among FLT3 inhibitors currently in development, in that it combines high potency and high kinase selectivity with favorable pharmacokinetic properties, and it is currently suggested as the most promising FLT3 targeting TKI [78]. It showed promising results already in a phase 1 study [61], and the optimism has remained high until now. In a phase 2 study, quizartinib was administered as monotherapy in 333 relapsed or refractory AML patients. The most frequent experienced treatment-related adverse events were nausea, anemia, QT interval prolongation, vomiting, febrile neutropenia, diarrhea, and fatigue. Quizartinib seemed to reduce blasts in both FLT3-wild-type as well as FLT3-ITD positive patients, though more efficiently in FLT3-ITD positive patients, and the overall clinical response rates were high, however few complete remissions were seen [63,64]. Results from a phase 2 study assessing quizartinib as monotherapy comparing two different dosing scheduled confirm a high degree of antileukemic activity of quizartinib in FLT3-ITD positive AML patients with half of included patients achieving a hematological response, and as many as 33% of patients were successfully bridged to hematological stem cell transplantation [62]. A pilot establishing that quizartinib safely can be combined with chemotherapy demonstrated a 79% complete remission rate in evaluable patients [65]. Quizartinib in combination with cytarabine and etoposide was also assessed in 18 pediatric AML patients, either with relapsed or refractory disease. Eight of the patients were FLT3-ITD positive and of the six assessed four patients achieved complete remission or complete remission with incomplete platelet recovery [66]. 

## 3. Discussion

Current clinically established therapy regimes for AML mainly fail to achieve durable responses due to high relapse rates associated with development of drug resistance. Those patients who harbor a constitutively activating FLT3-ITD mutation have particular poor initial therapy response, high relapse rate, and inferior overall survival. Optimism was substantial when the therapeutic principle based on inhibition of FLT3 emerged, and multiple compounds have been developed and tested. Eight of the TKIs investigated in clinical trials have here been presented and the results of their clinical efficacy compared, including the first-generation agents; lestaurtinib, linifanib, midostaurin, semaxanib, sorafenib, sunitinib and tandutinib, as well as the second-generation TKI quizartinib. Though the various compounds diverge in degree of treatment responses as well as character and seriousness of adverse effects, they are generally well tolerated with less toxic effects than conventional chemotherapy provided in high or intermediate dose levels. However, TKIs seems only to induce modest clinical effects, including partial and transient responses, usually only in peripheral blasts. 

There may be several reasons for why AML patients with FLT3-ITD mutations do not respond to treatment as anticipated. In the majority of the clinical trials, the treatment with TKIs were limited to patients with relapsed or refractory disease, or to patients not eligible for conventional treatment. The experience from this selected patient group might not be applicable to the group of newly diagnosed or younger AML patients. 

*In vivo* inhibition of FLT3 autophosphorylation seems to be greatly associated with remission rate, and insufficient prolonged inhibitory drug levels might be another reasons for treatment failure [79]. Clinical response occurred in patients who sustained plasma FLT3 inhibitory activity and had an inherent sensitivity of blasts to the cytotoxic effects of lestaurtinib [44]. Several second generation TKIs, in addition to quizartinib, are under development, offering improved pharmacodynamic and pharmacokinetic properties including increased potency and selectivity towards FLT3-mutated cells [80]. VX-322 [81], BPR1J-097 [82], TT-3002 [83,84], AKN-028 and AKN-032 [85,86] are examples of novel FLT3 inhibitors that are showing promising *in vitro* and *in vivo* antileukemia activities. 

Many of the compounds tested in clinical trials gave an initial response but of short duration before relapse, indicating development of resistance. Acquired point mutations in the molecular target of FLT3 in response to TKI treatment, precluding the drug from adequate binding appears to be an important mechanism in this process [87,88]. Aberrant activation of alternate growth and viability pathways is yet another possible mechanism for acquired resistance [89,90,91]. 

Unexpectedly, it was not so easy to predict who would benefit from the treatment as assumed. Not all FLT3-ITD positive patients responds to TKI therapy, while on the contrary some FLT3 wild-type patients seem to benefit from TKI treatment. Biomarkers predictive of therapy response are warranted. It is suggested that quizartinib does not induce complete remission, but decrease blast numbers with presence of dysplastic changes in the bone marrow [92]. In a small study of quizartinib-treated AML patients examined by mass spectrometric super-SILAC of phospho-protein, the team of Hubert Serve have indicated that a profile of four proteins may determine responders of quizartinib independent of FLT3-ITD [93]. This proposes phophoprotein profiling in prediction of therapy response, and may be transferred to a clinical diagnostics assay format like flow cytometric analysis of intracellular phosphoproteins [94].

Although FLT3 is a well-characterized oncoprotein in AML, and its role as an important player in AML leukemogenesis established, our knowledge of the normal and pathologic FLT3 signaling network may still be inadequate for identification of the most effective therapeutic approach, as there are many aspects of the mutation we do not fully comprehend. 

An initial concern in the study of FLT3 in AML was the heterogeneity of the mutations, both the difference between point mutations and ITDs and within the group of ITDs. The mutation can appear in various lengths in the same patient, either simultaneously or over time, and in response to intensive chemotherapy the mutation can appear in previously FLT3-ITD negative patients, it can disappear or dramatically change at relapse [95,96,97,98,99]. The size of the ITD has been reported as a prognostic marker, with patients with a insertion of 48–60 base pairs seems to have worse outcome compared to patients with shorter or longer insertions in one study [100], and with increasing size as a marker for poor outcome in another [101]. High mutational load measured by a high FLT3-ITD/FLT3wt ratio, indicative of loss of heterozygosity, has been associated with inferior outcome [102,103], as well as the site of the ITD insertion, with insertions within the tyrosine kinase domain-1 conferring unfavorable prognosis [104]. Also, the number of FLT3-ITD mutations affects disease outcome [105].

Methodological advances have recently shed further light to the complex interplay of events that contribute to AML leukemogenesis [106]. In addition to formerly well-characterized frequent cytogenetic lesions, next generation sequencing of AML patient material has revealed 23 recurrently mutated genes probable to be involved in AML pathogenesis. Based on patterns of co-occurring and mutually exclusive genetic lesions probable biological co-operations driving disease progression are emerging [19,107]. Additionally, mapping of variant allele frequencies makes it possible to assess the intra-tumor clonal hierarchy, while temporal assessment of leukemic cell populations makes it achievable to determine the clonal evolution and the sequential order of acquisition of somatic mutation during disease development and progression, from pre-leukemic hematopoietic stem cells to AML blasts [108,109,110]. Accumulating evidence indicate that mutations in the FLT3 gene are disease promoting rather than disease initiating events [111,112], and that mutant FLT3 cooperates with other oncogenes and aberrantly regulated proteins associated with AML, e.g., NPM1, DNMT3A [19], NUP98/NSD1 [113] DEP-1, PML-RAR and AXL [114,115,116]. The potency of the TKI alone may consequently not be the best measure for the antileukemic effect, and a multi-targeted therapeutic approach may rather be of potential clinical benefit, combining agents targeting cooperative lesions, inhibitors of alternate pathways, or targeting downstream signaling molecules. The superior efficiency of sorafenib compared to other inhibitors supports this theory, as the effect might be a result of sorafenib’s ability to suppress the activity of multiple pathways [53]. The pathways that most frequently are activated, PI3K/AKT/mTOR and RAS/RAF/MEK/ERK, may be feasible to target with a combined inhibitor approach. Effective AKT/mTOR inhibitors and MEK/ERK inhibitors are in clinical trials [117,118]. FLT3-ITD is additionally shown to accumulate in the endoplasmic compartment of the cell [119], and may form intracellular signaling protein complexes that represent a different signaling context compared to transmembrane FLT3 signaling [120], that might also be important to take into consideration.

The nature of internal tandem duplication mimic damages in DNA repair caused by anthracyclines [121,122]. Clinical studies with increasing doses of daunorubicin suggested that this dose escalation was not beneficial in FLT3-ITD patients [123]. Additionally, a retrovirally induced mouse leukemia model comprising FLT3-ITD indicated that the FLT3-ITD responded to cytarabine but not anthracycline in a p53 dependent manner [124]. Additionally, excessive receptor tyrosine kinase activity has been associated with increased endogenous DNA damage [125], and FLT3-ITD is associated with high redox activity in the leukemic cells, also related to increased DNA damage [126]. In the discussion of driver and passenger role for FLT3-ITD, it is difficult to neglect the possibility that leukemia with FLT3-ITD may be created through a fundamental genomic instability. This genomic instability may be targeting the FLT3 gene due to structural DNA features through the myelopoiesis when FLT3 expression is modulated as function of differentiation. This predisposition of FLT3-ITD may make these leukemia cells particularly vulnerable for anthracyline therapy, generating more FLT3-ITD mutations when exposed for this topoisomerase II inhibitor. Together, these observations indicate that FLT3-ITD positive patients does not benefit from anthracycline therapy. If this is correct, a dramatic change in current AML therapy needs to be undertaken, since all current induction therapy include anthracycline. 

Together, the proven high relapse rate in FLT3-ITD and the emerging speculations in an underlying mutational vulnerability in the FLT3 gene should spur investigators to develop non-genotoxic therapy in particular for FLT3-ITD positive AML patients. 

## 4. Concluding Remarks

A fundamental question that remains unanswered is whether the fairly modest clinical activity of first generation FLT3 inhibitors can be improved through the second generation of TKIs, offering better pharmacodynamic and pharmacokinetic properties, or if the potential benefits of FLT3 inhibitors are essentially inadequate. We are still awaiting results from ongoing clinical trials investigating various combinations of TKIs in different subgroups of AML patients. The preliminary conclusion concerning the agents investigated is that their therapeutic efficiency is limited when administered in monotherapy. It seems like the FLT3 inhibitors currently in clinical trials will have to be used in conjunction with established treatment or in combination with additional targeted therapeutics to ultimately improve outcomes in AML patients with FLT3-ITD mutations. It is also to be decided in which phase of the treatment is should be used; as part of first line induction therapy, as consolidation or post-remission treatment or in a relapse or refractory setting. 

If presence of FLT3-ITD is a marker for less effective anthracycline therapy, we will need to perform a difficult switch to alternative induction regimes in these patients. Future trials should explore targeting of downstream FLT3 signaling, particular signaling unique for FLT3-ITD, and with a clear strategy for blocking bypass mechanisms that may cause TKI resistance. These alternative strategies of signal transduction therapy may be tested in future trials incorporating *in vitro* leukemic resistance screens [127,128] determining the value of functional genomics in individualized therapy strategies [126,127]. 
